# Household knowledge, perceptions and practices of mosquito larval source management for malaria prevention and control in Mwanza district, Malawi: a cross‐sectional study

**DOI:** 10.1186/s12936-021-03683-5

**Published:** 2021-03-17

**Authors:** Mphatso Kamndaya, Dumisani Mfipa, Kingsley Lungu

**Affiliations:** 1 Department of Mathematics and Statistics, School of Applied Sciences, University of Malawi, The Polytechnic, Private Bag 303, Blantyre 3, Malawi; 2 Department of Environmental Health, School of Applied Sciences, University of Malawi, The Polytechnic, Private Bag 303, Blantyre 3, Malawi

**Keywords:** Larval source management, Knowledge, Perceptions, Practices

## Abstract

**Background:**

Mosquito larval source management (LSM) is a key outdoor malaria vector control strategy in rural communities in sub-Saharan Africa. Knowledge of this strategy is important for optimal design and implementation of effective malaria control interventions in this region. This study assessed household knowledge, perceptions and practices of mosquito LSM methods (draining stagnant water, larviciding, clearing grass/bushes and clean environment).

**Methods:**

A cross-sectional design was used whereby 479 households were selected using two-stage sampling in Mwanza district, Malawi. A household questionnaire was administered to an adult member of the house. Respondents were asked questions on knowledge, perceptions and practices of mosquito LSM methods. Multivariable logistic regression model was used to identify factors associated with high-level knowledge of mosquito LSM methods.

**Results:**

Majority of the respondents (64.5%) had high-level knowledge of mosquito LSM methods. Specifically, 63.7% (200/314) had positive perceptions about draining stagnant water, whereas 95.3% (223/234) practiced clean environment for malaria control and 5.2% had knowledge about larviciding. Compared to respondents with primary education, those with secondary education were more likely, whereas those without education were less likely, to have high-level knowledge of mosquito LSM methods (AOR = 3.54, 95% CI 1.45–8.63 and AOR = 0.38, 95% CI 0.23–0.64, respectively). Compared to respondents engaged in crop farming, those engaged in mixed farming (including pastoralists) and the self-employed (including business persons) were more likely to have high-level knowledge of mosquito LSM methods (AOR = 6.95, 95% CI 3.39–14.23 and AOR = 3.61, 95% CI 1.47–8.86, respectively). Respondents living in mud-walled households were less likely to have high-knowledge of mosquito LSM methods than those living in brick-walled households (AOR = 0.50, 95% CI 0.30–0.86).

**Conclusions:**

A high-level knowledge of mosquito LSM methods was established. However, when designing and implementing this strategy, specific attention should be paid to the uneducated, crop farmers and those living in poor households.

**Supplementary Information:**

The online version contains supplementary material available at 10.1186/s12936-021-03683-5.

## Background

Malaria remains a global health challenge due to its high disease burden [1]. Sub-Saharan Africa (SSA) region has high temperatures and rainfall climate that present favourable breeding environment for malaria-transmitting anopheline mosquitoes [1] such that malaria is the leading cause of death and illness in SSA [2]. Over 15% of hospital admissions in this region are due to malaria [3]. Almost all Malawians are at risk of malaria [4]. Malaria is among diseases that lead to years-lost-to-disability (YLD); 30% of outpatient visits are due to malaria in Malawi [5]. Over 40% of hospital admissions are among children under 5 years old in Malawi [6]. Most malaria control interventions ignore the main culprit: outdoor transmission [7]. Thus, an effective policy setting is critical to fight malaria in the wake of financial constraints and increased resistance of *Plasmodium falciparum* to anti-malarial drugs [2]. The World Health Organization (WHO) established a Malaria Policy Advisory Committee (MPAC) to set evidence-informed policies for implementation to control and eliminate malaria in WHO member states [8], and MPAC attributed the worldwide achievements in malaria control over the past decade to major investments in vector control [9]. Malawi spends much financial resource on indoor transmission interventions [10] but outdoor mosquito biting remains a challenge in SSA countries, including Malawi [11]. Mosquito larval source management (LSM) methods would be useful to eliminate the main malaria vectors in SSA: *Anopheles gambiae* and *Anopheles arabiensis* [11]. Limited community participation, however, has been identified as the main barrier to LSM in Malawi [12]. Evidence suggests that the strategy is cheap and easily implementable at household and community levels [11]. This study assessed household knowledge, practices and perceptions of mosquito LSM methods to guide the design and implementation of effective malaria control interventions in rural Malawian communities.

## Methods

### Study design

**Fig. 1:**
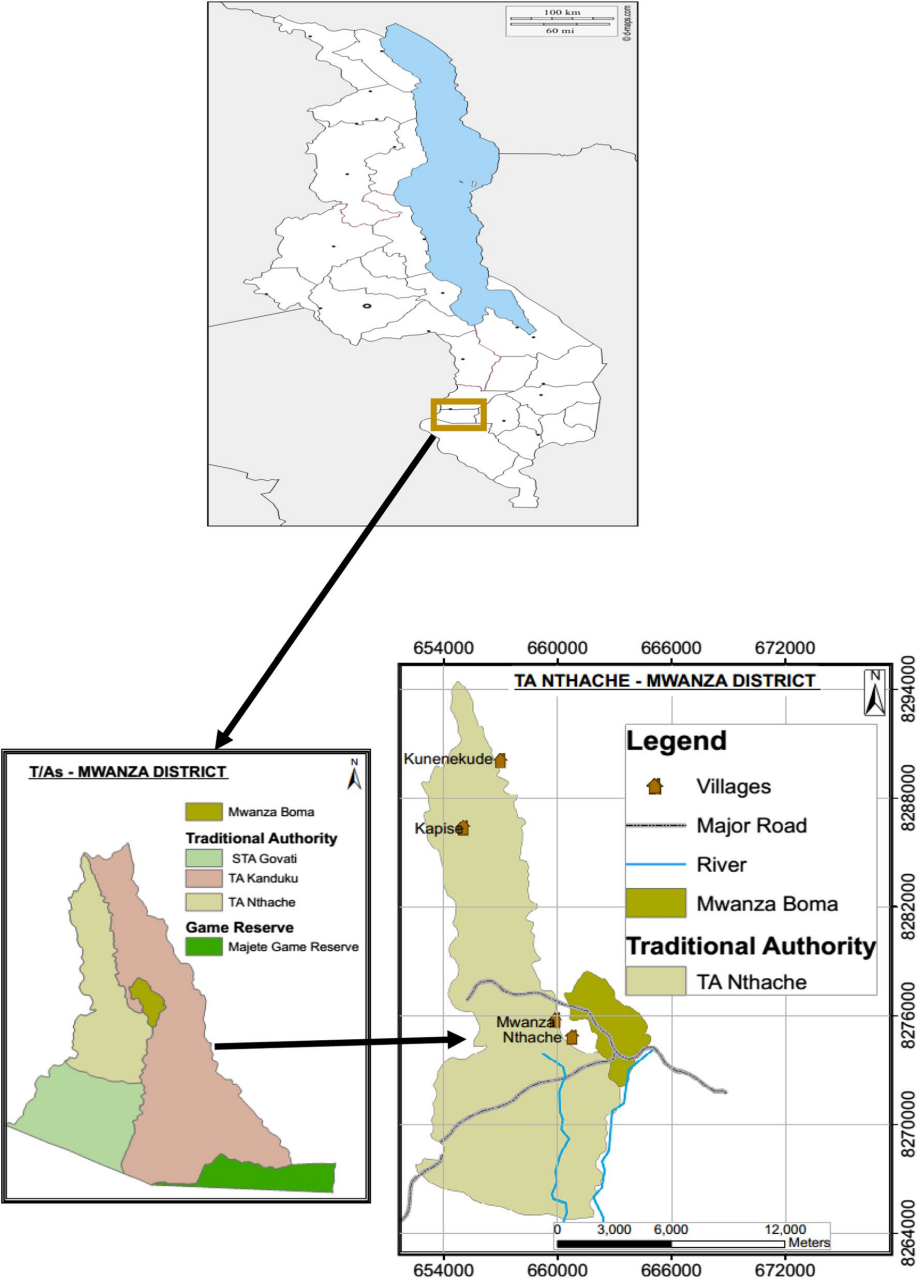
Map of Traditional Authority Nthache in Mwanza district, Malawi

A cross-sectional design was conducted in Nthache area in Mwanza district, Malawi (Fig. 1).

### Sampling

A sample size of 497 households was calculated using Cochran formula [13]. A non-response rate of 10% and a proportion of 30% with knowledge of mosquito LSM methods were assumed based on a similar rural Kenyan study [14]. A design effect of 1.4 was calculated based on a cluster size, *b* of 15 and a rate of homogeneity of 0.025 [15]. Level of precision was set at 0.05. Sample size was adjusted to 500 to maintain an equal number of 20 households per cluster. Some 479 respondents were interviewed as 21 respondents were unavailable. A two-stage cluster random sampling procedure was used to select households for this study.

In Stage 1, 25 clusters were randomly selected from 43 villages that form Nthache area, using probability proportional to population size. Each selected village formed a cluster or two clusters depending on population size (Additional file 1). Prior to random selection of clusters, a sampling interval (SI) was calculated after dividing the total population in the study area (33,870) into 25 clusters. Cumulative population sizes for the villages were calculated by size of the population for each village [13]. A range was developed for each village according to its cumulative population size. To determine clusters, a number 52 was randomly selected between 1 and a calculated SI of 1,354.8. The village with a range that contained the number 52 was identified as the first cluster. The SI was then added to 52 to determine the second cluster. This process continued as SI was added to the immediate calculated result until 25 clusters were identified [13].

In stage 2, village registers from the sampled clusters were used to assign households with identification numbers. Small pieces of paper with identification numbers were put in a pot, from which 20 households were selected per cluster.

### Research variables

A validated household questionnaire was used to collect data. Respondents were asked questions on socio-demographic characteristics, knowledge, perceptions, and practices related to specific mosquito LSM methods. An adult member in the household was interviewed (> 18 years old). In child-headed households (< 18 years-old), the heads were interviewed. An adult member from the sampled household who had consented to respond to survey questionnaires was included.

Gender of the respondent was classified and coded as female, 1 and male, 2. Data on age was collected as a continuous variable and categorized as well as coded into a binary variable of ≤ 35 years: 1, > 35 years: 2. Education status was classified and coded as primary: 1, secondary: 2, none: 3, informal/pre-primary: 4. Marital status was classified and coded as married: 1, single: 2, widowed: 3, divorced/separated: 4. Pregnancy status was classified and coded as being not-pregnant: 1, pregnant: 2. Occupation was classified and coded as crop farming: 1, mixed farming/pastoralist: 2, business/self-employed: 3, unemployed/student: 4, employed: 5, housewife: 6, other: 7. Household ownership was classified and coded as owned: 1, rented: 2. Household floor was categorized and coded as natural/earth: 1, cement/tiles: 2. Household roof was classified and coded as grass/thatch: 1, iron sheets: 2. Wall type was classified and coded as brick wall: 1, mud wall: 2. Energy used was classified and coded as firewood: 1, charcoal: 2.

Level of knowledge was measured as a binary variable (1-high-level and 0-low-level). A scoring system known as knowledge score was developed to assess the level of knowledge. To score full points (4 points), respondents had to mention four methods: draining stagnant water (1 point), larviciding (1 point), clearing grass/bushes (1 point), and clean environment (1 point). A mean score of 1.73 was calculated. Respondents with scores above the mean were deemed to have high knowledge whereas those below it were deemed to have low knowledge of mosquito LSM methods [16]. Respondents were asked to mention specific mosquito LSM methods. They could provide multiple responses from this list: draining stagnant water, larviciding, clearing grass/bushes, clean environment. Those who stated correct answers were deemed as having knowledge whereas those with incorrect/unstated answers as not having knowledge. The responses were coded as 1: having knowledge, 0: not having knowledge. Respondents were asked to mention one specific mosquito LSM method they perceived as the most effective for malaria control among those they initially expressed knowledge of. The responses were coded as 1: positive perceptions, 0: no positive perceptions. Respondents were asked to mention specific mosquito LSM methods they practice for malaria control they initially expressed knowledge of. The responses were coded as 1: practiced, 0: not practiced (Additional file 2).

### Statistical analysis

Descriptive statistics related to a range of socio-demographic characteristics, knowledge, perceptions, and practices of mosquito LSM methods were calculated. Logistic regression model using enter method was used to identify factors associated with high-level knowledge of mosquito LSM methods. The model adjusted for gender and selected socio-demographic variables (excluding pregnancy status). All statistical analyses used SPSS version 18.

### Ethical considerations

Ethical approval for this study was obtained from the Malawi National Health Science Research Committee (NHSRC) (Approval number: 2158).

## Results

A total of 479 respondents, aged 16–81 years, participated in this study. Majority of them (64.5%) showed high-level knowledge of mosquito LSM methods (Table 1). Table 2 shows household knowledge, perceptions and practices regarding specific mosquito LSM methods. A small proportion of the respondents (5.2 %) had knowledge of larviciding. About 65.6% of the respondents had knowledge regarding draining stagnant waters as a malaria control method. Some 63.7 % (200/314) of the respondents had positive perceptions regarding draining stagnant waters while 39.8% (101/254) had positive perceptions regarding clearing grass/bushes as the most effective method for malaria control. It was observed that 90.2% (229/254) and 95.3% (223/234) of the respondents practiced malaria control by clearing grasses/bushes and clean environment around the home, respectively.

**Table 1:** Socio-demographic factors of respondents and their levels of knowledge regarding mosquito LSM methods in Nthache area, Mwanza district

Characteristics	N = 479 n (%)
Sex	
Male	140 (29.2)
Female	339 (70.8)
Age-group (years)^a^	
≤ 35 years-old	273 (57.0)
> 35 years-old	202 (42.2)
Marital status	
Married	379 (79.1)
Single	22 (4.6)
Widowed	41 (8.6)
Divorced/separated	37 (7.7)
Education status	
Primary	307 (64.1)
None	111 (23.2)
Secondary	56 (11.7)
Informal/pre-primary	5 (1.0)
Pregnancy status (women-of-child-bearing-age between 15–49 years-old)	N = 276
Not-pregnant	258 (93.5)
Pregnant	18 (6.5)
Occupation	
Crop farming	286(59.7)
Mixed-farming (crop and livestock)/pastoralist	93 (19.4)
Business/self-employed	42 (8.8)
Employee	25 (5.2)
Housewife	22 (4.6)
Unemployed/student	7 (1.5)
Other	4 (0.8)
Household ownership	
Owned	466 (97.3)
Rented	13 (2.7)
Household floor	
Natural/earth	398 (83.1)
Cement/tiles	81 (16.9)
Household roof	
Grass/thatch	292 (61.0)
Iron-sheets	187 (39.0)
Household wall	
Brickwall	344 (71.8)
Mudwall	135 (28.2)
Household energy used	
Firewood	433 (90.4)
Charcoal	46 (9.6)
Levels of knowledge regarding mosquito larval source management methods	
High	309 (64.5)
Low	170 (35.5)

^a^Age of 4 (0.8 %) respondents were missing in the dataset

**Table 2:** Knowledge, perceptions and practices regarding specific mosquito LSM methods among respondents in Nthache area, Mwanza district

Household knowledge	N = 479 n (%)
Knowledge about draining stagnant water	314 (65.6)
Knowledge about larviciding	25 (5.2)
Knowledge about clearing grass/bushes	254 (53.0)
Knowledge about clean environment	234 (48.9)
Household practices	
Practices regarding draining stagnant water	N = 314 270 (86.0)
Practices regarding larviciding	N = 25 8 (32)
Practices regarding clearing grass/bushes	N = 254 229 (90.2)
Practices regarding clean environment	N = 234 223 (95.3)
Household perceptions	
Positive-perceptions regarding draining stagnant water	N = 314 200 (63.7)
Positive-perceptions regarding larviciding	N = 25 8 (32)
Positive-perceptions regarding clearing grass/bushes	N = 254 101 (39.8)
Positive-perceptions regarding clean environment	N = 234 100 (42.7)

Table 3 shows the results of factors associated with high-level knowledge of mosquito LSM methods among respondents in Nthache, Mwanza. Compared to respondents with primary education, those with secondary education were more likely, whereas those without education were less likely, to have high-level knowledge of mosquito LSM methods (AOR = 3.54, 95% CI 1.45–8.63, P = 0.005 and AOR = 0.38, 95% CI 0.23–0.64, P = 0.000, respectively). Compared to respondents engaged in crop farming, those engaged in mixed farming (including pastoralists) and the self-employed (including business persons) were more likely to have high-level knowledge of mosquito LSM methods (AOR = 6.95, 95% CI 3.39–14.23, P = 0.000 and AOR = 3.61, 95% CI 1.47–8.86, P = 0.005, respectively). Respondents living in mud-walled households were less likely to have high-level knowledge of mosquito LSM methods than those living in brick-walled households (AOR = 0.50, 95% CI 0.30–0.86, P = 0.011).

**Table 3:** Factors associated with high-level knowledge regarding mosquito LSM methods among respondents in Nthache area, Mwanza district

Factors	Categories	High-level knowledge				
		COR	95% CI	AOR	95% CI	P-value
Sex	Female	1.00	Ref	1.00	Ref	
	Male	1.35	0.89–2.06	1.08	0.65–1.77	0.772
Marital status	Married	1.00	Ref	1.00	Ref	
	Single	0.85	0.35–2.08	0.43	0.13–1.38	0.155
	Widowed	0.38	0.20–0.73	0.74	0.33–1.67	0.468
	Divorced/separated	0.71	0.36–1.42	0.89	0.42–1.89	0.757
Education status	Primary	1.00	Ref	1.00	Ref	
	Secondary	3.38	1.48–7.73	3.54	1.45–8.63	*0.005*
	None	0.40	0.25–0.62	0.38	0.23–0.64	*0.000*
	Informal/pre-primary	0.73	0.12–4.41	0.92	0.13–6.86	0.939
Occupation	Crop farming	1.00	Ref	1.00	Ref	
	Mixed-farming/pastoralist	5.55	2.83–10.85	6.95	3.39–14.23	*0.000*
	Business/self-employed	3.72	1.60–8.66	3.61	1.47–8.86	*0.005*
	Unemployed/student	0.56	0.12–2.54	0.57	0.10–3.22	0.523
	Employed	0.95	0.42–2.16	0.88	0.36–2.11	0.768
	Housewives	0.62	0.26–1.48	0.58	0.22–1.52	0.267
	Other	0.25	0.03–2.41	0.32	0.03–3.62	0.359
Household wall	Brickwall	1.00	Ref	1.00	Ref	
	Mudwall	0.52	0.34–0.78	0.50	0.30–0.86	*0.011*
Household floor	Natural/earth	1.00	Ref	1.00	Ref	
	Cement/tiles	1.99	1.15–3.47	1.30	0.65–2.60	0.466
Household roof	Grass/thatch	1.00	Ref	1.00	Ref	
	Iron sheets	1.62	1.10–2.41	1.17	0.67–2.04	0.590

Multivariate model adjusted for sex, marital status, education status, occupation, household wall, household floor and household roof. Age-group (years), pregnancy status, household ownership and energy type were not significant at bivariate level

Italic values indicate significance of p value (p ≤ 0.05)

## Discussion

The purpose of this study was to assess knowledge, perceptions and practices of mosquito LSM methods to guide the design and implementation of effective malaria control interventions in rural Malawian communities. A high-level knowledge of mosquito LSM methods was observed in the study population. This finding was in contrast with a Tanzanian study that reported few respondents in the community had knowledge of the usefulness of mosquito LSM methods in malaria control [17]. This was due to failure by policy makers and implementers to prioritize methods in Tanzanian rural areas [17]. This study’s findings further showed that respondents without education were less likely to have high-level knowledge of mosquito LSM methods than those with primary education, a finding similar to a Cameroonian study that showed that literate people had a better understanding of malaria messages through formal education and media [18]. However, a Ghanaian study showed no significant association between education level and knowledge of malaria prevention [19]. Hence, non-formal and informal education was recommended for malaria control-related health promotion interventions. There is need to raise community awareness of mosquito LSM methods among non-literate people. According to Mukabana et al., community-based educational programmes for larval control should be communicated through personal interaction based on learning-by-doing [17]. These educational programmes have the potential to change the health status through new knowledge gained, changing perceptions, gaining and practicing new skills and behaviour [20]. A lack of incentives, labour-intensiveness and the time-demanding nature of LSM activities were barriers to community participation in Malawi [12].

This study showed a very low proportion of respondents with knowledge of larviciding as a malaria control method, similar to a study in east-central Tanzania that showed that majority of respondents were unaware of larviciding [17]. This was attributed to the fact that larviciding programmes were limited and restricted to urban areas, so that rural people were unaware of it [17]. There is need for health promotion interventions in rural areas to include larviciding. The present study’s results showed that close to two-thirds of respondents had knowledge of draining stagnant water as a malaria control method. This was comparable to an Ethiopian study that found that 84.2% of the respondents knew of draining stagnant water as a malaria control method [21]. Both results indicate inadequate knowledge among respondents regarding draining stagnant water as a malaria control method. This calls for increased awareness of this method in SSA where malaria is prevalent, including Malawi.

Compared to respondents engaged in crop farming, the self-employed (including business persons) and those engaged in mixed farming (including pastoralists) were more likely to have high-level knowledge of mosquito LSM methods. Further, a study in Tanzania observed that some aagricultural activities generate larval habitats whereas others control malaria [22]. Mboera et al*.* observed limited knowledge of human activities that contribute to malaria transmission among rural Tanzanian farmers [22]. This could explain why crop farmers in this Malawi study were unlikely to have high-level knowledge of mosquito LSM methods. Special consideration should be given to crop farmers when designing and implementing mosquito LSM methods programmes.

Imbahale et al. developed a basic socio-economic indicator based on information on the type of house owned [14]. The lowest indicator was associated with traditional houses (grass-thatched and mud-walled) [14]. The findings in this Malawian study suggested that respondents living in mud-walled households were less likely to have high-level knowledge of mosquito LSM methods than those living in brick-walled households. However, an American study observed that respondents from low-income households were more highly motivated to control mosquitoes than those from high-income households [23]. Hence, it is necessary to target both low- and high-income households with malaria prevention and control-related health promotion interventions to improve knowledge, perceptions and practices. Here, this study observed that few respondents had positive perceptions for larviciding as a malaria control method. Acceptability of larviciding was affected by poor perceptions about its safety in east-central Tanzania [17]. Safety in larviciding in rural areas should be prioritized.

As to limitations, firstly, associations derived from cross-sectional studies fell short of establishing temporal relationship between factors and outcomes of the study. Secondly, it is possible that those individuals with knowledge of mosquito LSM methods may not have been adult members of households that answered the questionnaires. Thirdly, data collection was done during the rainy season when malaria was prevalent, and when respondents could have been exposed to messages on mosquito LSM methods that influenced their responses. It is, therefore, important to generalize these results with caution as they may not necessarily reflect knowledge, perceptions and practices of mosquito LSM methods in Malawi.

## Conclusions

This study established a high-level knowledge of mosquito LSM methods. Besides education, a well-known predictor of high-level knowledge of mosquito LSM methods, occupation and socio-economic status were also identified as predictors. Therefore, the uneducated, crop farmers and poor households should be considered when designing and implementing this strategy. Larviciding was the least known mosquito LSM method, and it is therefore important to promote awareness and practice of larviciding in rural communities. Positive perceptions for clearing grass/bushes and larviciding were low. Health workers should focus on targeted health promotion interventions regarding mosquito LSM methods.

## Supplementary Information

**Additional file 1.** A table of calculations of cluster sampling using probability proportional to population size.

**Additional file 2.** An SPSS data file.

## Data Availability

The dataset is available on reasonable request to authors.

## Abbreviations

AOR: Adjusted odds ratio
b: Cluster size
CI: Confidence interval
COR: Crude odds ratio
LSM: Larval source management
MPAC: Malaria Policy Advisory Committee
NHSRC: National Health Sciences Research Committee
roh: Rate of homogeneity
NIMR: National Institute for Medical Research
SI: Sampling interval
WHO: World Health Organization

## References

[1] Chanda E, Mzilahowa T, Chipwanya J, Mulenga S, Ali D, Troell P (2015). Preventing malaria transmission by indoor residual spraying in Malawi: grappling with the challenge of uncertain sustainability. Malar J.

[2] WHO. World Malaria Report 2011. Geneva: World Health Organization; 2011.

[3] Robert V, Macintyre K, Keating J, Trape J-F, Duchemin J-B, Warren M (2003). Malaria transmission in urban sub Saharan Africa. Am J Trop Med Hyg.

[4] Malawi Ministry of Health (2012). Malawi Health Management Information System (HMIS).

[5] Ministry of Health [MoH]. Malawi Health Sector Strategic Plan II 2017–2022: Towards Universal Coverage. Lilongwe: Ministry of Health; 2017.

[6] Ministry of Health [MoH]. Malawi National Health Communication Strategy, 2015–2020. Lilongwe: Ministry of Health; 2015.

[7] Govella NJ, Ferguson H (2012). Why use of interventions targeting outdoor biting mosquitoes will be necessary to achieve malaria elimination. Front Physiol.

[8] D’Souza B, Newman R (2012). Strengthening the policy setting process for global malaria control and elimination. Malar J.

[9] WHO Malaria Policy Advisory Committee and Secretariat (2013). Malaria Policy Advisory Committee to the WHO: conclusions and recommendations of September 2013 meeting. Malar J.

[10] Fillinger U, Lindsay S (2011). Larval source management for malaria control in Africa: myths and reality. Malar J.

[11] WHO. Larval source management: a supplementary measure for malaria vector control: an operational manual. Geneva: World Health Organization; 2013.

[12] Gowelo S, McCann RS, Koenraadt CJ, Takken W, van den Berg H, Manda-Taylor L (2020). Community factors affecting participation in larval source management for malaria control in Chikwawa District. Southern Malawi Malar J.

[13] Sampling Methods and Sample Size Calculation for the SMART Methodology [Internet]. 2012. Available from: https://www.humanitarianresponse.info/sites/www.humanitarianresponse.info/files/documents/files/Sampling_Paper_June_2012.pdf

[14] Imbahale S, Fillinger U, Githeko A, Mukabana W (2010). An exploratory survey for malaria prevalence and people’s knowledge, attitudes and practices of mosquito larval source management for malaria control in western Kenya. Acta Trop.

[15] Knox SA, Chondros P (2004). Observed intra-cluster correlation coefficients in a cluster survey sample of patient encounters in general practice in Australia. BMC Med Res Methodol.

[16] Spjeldnaes A, Kitua A, Bloomberg B (2014). Education and knowledge helps combating malaria, but not degedege: a cross-sectional study in Rufiji. Tanzania Malar J.

[17] Mboera LE, Kramer RA, Miranda ML, Kilima SP, Shayo EH, Lesser A (2014). Community knowledge and acceptance of larviciding for malaria control in a Rural District of east-central Tanzania. Int J Environ Res Public Health.

[18] Kimbi HK, Nkesa SB, Ndamukong-Nyanga JL, Sumbele IU, Atashili J, Atanga MB (2014). Knowledge and perceptions towards malaria prevention among vulnerable groups in the Buea Health District. Cameroon BMC Public Health.

[19] Appiah-Darkwah I, Badu-Nyarko SK (2011). Knowledge of malaria prevention and control in sub-urban community in Accra. Ghana Int J Trop Med.

[20] Fertman CI, Allensworth DD (2010). Health promotion programs: from theory to practice.

[21] Abate A, Erko B (2013). Community knowledge, attitude and practice about malaria in low endemic setting of Shewa Robit Town, north eastern Ethiopia. BMC Public Health.

[22] Mboera LE, Mazigo HD, Rumisha SF, Kramer RA (2013). Towards malaria elimination and its implication for vector control disease management and livelihoods in Tanzania. Malar World J.

[23] Dowling Z, Armbruster P, LaDeau SI, De Cotiis M, Mottley J, Leisnham PT (2013). Linking mosquito infestation to resident socioeconomic status, knowledge and source reduction practices. EcoHealth.

