# Supplementary material for: Household knowledge, perceptions and practices of mosquito larval source management for malaria prevention and control in Mwanza district, Malawi: a cross‐sectional study
Source: Malar J. 2021 Mar 17;20:150. doi: 10.1186/s12936-021-03683-5 (PMC7967974; doi:10.1186/s12936-021-03683-5)
Supplement: Supplementary file 1 — Additional file 1. A table of calculations of cluster sampling using probability proportional to population size. [file 12936_2021_3683_MOESM1_ESM.docx]

## Cluster Sampling using Probability Proportional to Population Size

| No | Villages | Number of households | Population per village | Cumulative population | Range of cum. pop | Number of clusters per village | |
| --- | --- | --- | --- | --- | --- | --- | --- |
| 1 | Kapise | 302 | 1,404 | 1,404 | 1 -1,404 | | 1 |
| 2 | Ntasa | 105 | 488 | 1,892 | 1,405 - 1,892 | | 1 |
| 3 | Kachipanda | 97 | 450 | 2,342 | 1,893 -2,342 | | 0 |
| 4 | Faiti | 301 | 1,396 | 3,738 | 2,343 - 3,738 | | 1 |
| 5 | Biliwiri | 144 | 669 | 4,407 | 3,739 - 4,407 | | 1 |
| 6 | Kamwendo | 82 | 380 | 4,787 | 4,408 - 4,787 | | 0 |
| 7 | Chenedi | 40 | 184 | 4,971 | 4,788 - 4,971 | | 0 |
| 8 | Kasuza | 484 | 2,250 | 7,221 | 4,972 - 7,221 | | 2 |
| 9 | Nkulira I | 188 | 872 | 8,093 | 7,222 - 8,093 | | 0 |
| 10 | Kabango | 158 | 732 | 8,825 | 8,094 - 8,825 | | 1 |
| 11 | Kasapha | 392 | 1,823 | 10,648 | 8,826 -10,648 | | 1 |
| 12 | Mpandasoni | 416 | 1,930 | 12,578 | 10,649 - 12,578 | | 2 |
| 13 | Masokosa | 150 | 695 | 13,273 | 12,579 - 13,273 | | 0 |
| 14 | Mkwete | 267 | 1,240 | 14,513 | 13,274 - 14,513 | | 1 |
| 15 | Dzilima | 163 | 756 | 15,269 | 14,514 - 15,269 | | 1 |
| 16 | Chiwembu | 163 | 755 | 16,024 | 15,270 - 16,024 | | 0 |
| 17 | Njanjama I | 157 | 731 | 16,755 | 16,025 - 16,755 | | 1 |
| 18 | Makanani | 204 | 948 | 17,703 | 16,756 - 17,703 | | 1 |
| 19 | Kanyani | 378 | 1,754 | 19,457 | 17,704 - 19,457 | | 1 |
| 20 | Njanjama II | 77 | 357 | 19,814 | 19,458 –19,814 | | 0 |
| 21 | Kagulo II | 98 | 456 | 20,270 | 19,815 - 20,270 | | 0 |
| 22 | Chimlango | 96 | 447 | 20,717 | 20,271 - 20,717 | | 1 |
| 23 | Golowa | 140 | 652 | 21,369 | 20,718 - 21,369 | | 0 |
| 24 | Nkulira II | 142 | 658 | 22,027 | 21,370 -22,027 | | 1 |
| 25 | Chimbwinda | 157 | 729 | 22,756 | 22,028 - 22,756 | | 0 |
| 26 | Silota | 122 | 568 | 23,324 | 22,757 - 23,324 | | 1 |
| 27 | Kagulo I | 135 | 628 | 23,952 | 23,325 - 23,952 | | 0 |
| 28 | Nthache | 287 | 1,334 | 25,286 | 23,953 - 25,286 | | 1 |
| 29 | Kagonamwake | 145 | 675 | 25,961 | 25,287 - 25,961 | | 1 |
| 30 | Kapherana | 137 | 637 | 26,598 | 25,962 - 26,598 | | 0 |
| 31 | Ilemba | 127 | 588 | 27,186 | 26,599 - 27,186 | | 1 |
| 32 | Chikoleka | 83 | 385 | 27,571 | 27,187 - 27,571 | | 0 |
| 33 | Ndilire | 100 | 465 | 28,036 | 27,572 - 28,036 | | 0 |
| 34 | Galatiya | 118 | 549 | 28,585 | 28,037 - 28,585 | | 1 |
| 35 | Chiwambo | 135 | 626 | 29,211 | 28,586 - 29,211 | | 0 |
| 36 | Mangulenje | 74 | 345 | 29,556 | 29,212 - 29,556 | | 0 |
| 37 | Ng’onzo I | 127 | 590 | 30,146 | 29,557 - 30,146 | | 1 |
| 38 | Ng’onzo II | 136 | 633 | 30,779 | 30,147 - 30,779 | | 0 |
| 39 | Pfupa I | 138 | 643 | 31,422 | 30,780 -31,422 | | 1 |
| 40 | Pfupa II | 134 | 622 | 32,044 | 31,423 - 32,044 | | 0 |
| 41 | Nguteya | 128 | 593 | 32,637 | 32,045 - 32,637 | | 1 |
| 42 | Gunde | 122 | 593 | 33,230 | 32,638 - 33,230 | | 0 |
| 43 | Gwiremchira | 138 | 640 | 33,870 | 33,231 -33,870 | | 0 |
| Total | | 7,287 | 33,870 |  |  | | 25 |
