# Supplementary figures and images for: Household knowledge, perceptions and practices of mosquito larval source management for malaria prevention and control in Mwanza district, Malawi: a cross‐sectional study
Source: Malar J. 2021 Mar 17;20:150. doi: 10.1186/s12936-021-03683-5 (PMC7967974; doi:10.1186/s12936-021-03683-5)

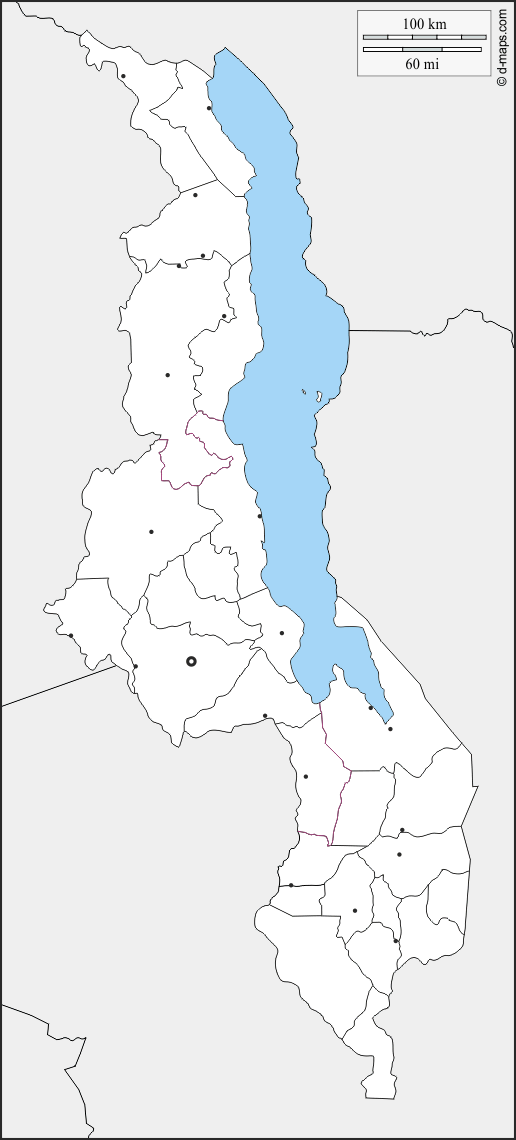


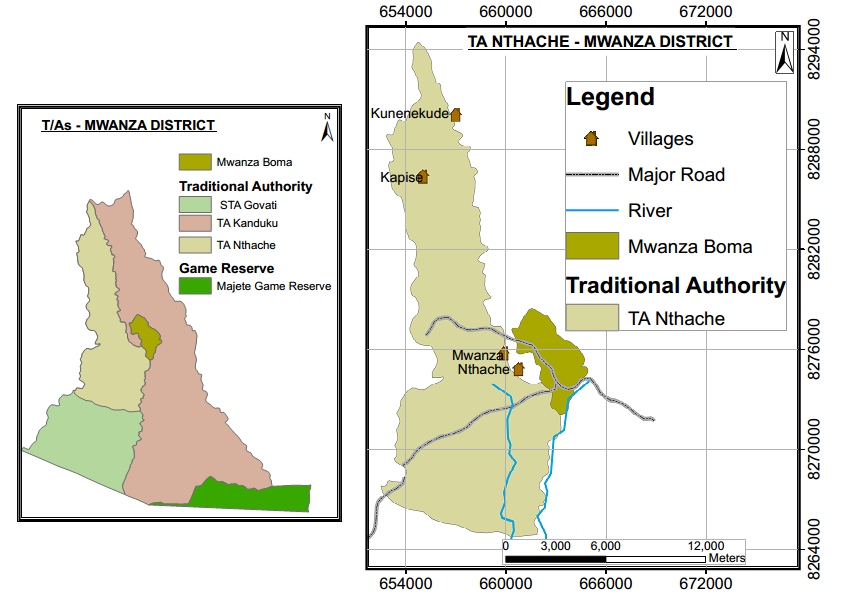

Supplement: Supplementary file 2 — Additional file 2. An SPSS data file. [file 12936_2021_3683_MOESM2_ESM.docx]
